# Findings from a pilot project to assess the feasibility of active tuberculosis case finding among seniors in rural Sichuan Province, China, 2017

**DOI:** 10.1371/journal.pone.0214761

**Published:** 2019-03-28

**Authors:** Canyou Zhang, Lan Xia, Jeanette J. Rainey, Yuan Li, Chuang Chen, Zhengyuan Rao, Jinchao Duan, Hongying Sun, Jie Cao, Ping Liu, Jun Cheng, Hui Zhang, Jianlin Wu, Lixia Wang

**Affiliations:** 1 National Center for Tuberculosis Control and Prevention, Chinese Center for Disease Control and Prevention, Beijing, China; 2 Sichuan Center for Disease Control and Prevention, Chengdu, Sichuan, China; 3 Division of Global Health Protection, United States Centers for Disease Control and Prevention, Beijing, China; 4 Mianyang Center for Disease Control and Prevention, Mianyang, Sichuan, China; 5 Jiangyou Center for Disease Control and Prevention, Jiangyou, Mianyang, Sichuan, China; Chinese Academy of Medical Sciences and Peking Union Medical College, CHINA

## Abstract

**Background:**

China has a substantial tuberculosis (TB) disease burden and an aging population. Seniors have a higher risk of developing TB disease compared to younger age groups. Active case finding (ACF) could help identify seniors with TB disease.

**Methods:**

From March to June 2017, we included ACF during annual physical check-ups for persons aged ≥ 65 years in Bayi, Sichuan Province. Seniors with clinical TB symptoms (i.e., cough lasting ≥ 2 weeks and/or hemoptysis) or one or more risk factors (e.g., previous TB disease, diabetes, and heavy alcohol consumption) were offered chest x-rays. We used acid-Fast Bacilli smear and solid culture laboratory testing for TB confirmation. We calculated the yield (i.e., cases identified among seniors screened) and cost per new each TB case detected. Focus group-interviews were conducted with health care workers and seniors to evaluate project acceptability. Participation rates and acceptability were used to assess feasibility.

**Results:**

Of the 2,393 seniors residing in Bayi, 2,049 (85.6%) were enrolled in the pilot project. Of these seniors, 794 (38.7%) presented with at least one TB risk factor and 74 (3.6%) had symptoms consistent with active TB disease. Three seniors (0.2%)–each presenting with at least one risk factor—were diagnosed with active TB. The project yielded 146 TB cases per 100,000 seniors screened; the cost per case detected was $4,897. Most workers supported ACF if additional resources and staff could be provided. Seniors appreciated the convenience of this integrated health service approach.

**Conclusions:**

Although the yield was lower than expected, ACF appeared feasible in Bayi. Targeting seniors with at least one known TB risk factor could help detect previously unidentified TB cases. However, similar projects in communities with a higher TB prevalence are needed to further evaluate the yield and required resources prior to implementation on a larger scale. Findings from our pilot project should be combined with data from these future ACF projects to improve TB screening criteria.

## Background

Although the number of newly diagnosed tuberculosis (TB) cases has declined substantially in China since 2009 [1], the country continues to experience a substantial TB disease burden with an estimated 889,000 incident cases per year (or 9% of new cases globally) [2]. Ensuring accurate and timely diagnosis and reporting of TB cases remains challenging, particularly in rural areas. Seniors–defined as persons over 65 years of age–are at a higher risk for TB compared to the other age groups in China. This is primarily due to a combination of changes in immune function, risk behaviors (e.g., tobacco use), and poor nutrition [3–5]. Despite this increased risk, China’s Fifth National TB Survey found that almost 40% of senior pulmonary TB patients did not present with typical symptoms (chronic cough or hemoptysis), and more than 50% did not actively seek medical care for their disease [6]. Assuming current control strategies and projected demographic changes, reactivation of TB in the elderly population will likely continue to produce new cases in China unless identified and treated effectively [7]. In 2014, China’s population over 65 years of age was estimated to represent at least 10% of the total population [8], leading health officials to prioritize active TB case-finding among seniors.

In 2009, China initiated a free annual physical check-up program for seniors as part of the Basic Public Health Service Package [9]. Each year, township health centers (in rural areas) or community health service centers (in urban areas) organize health management services that include evaluation of lifestyle and health status, physical examination, auxiliary examination, and health guidance. Findings from these check-ups are added to the residents’ health records. This service package was expanded to include assessments for suspected TB in 2015. The China National Health and Family Planning Commission (NHFPC) issued “The Standards of TB Management in Basic Public Health Service” to instruct primary healthcare workers (HCWs) to refer patients with TB symptoms to assigned healthcare facilities for TB, conduct health education for TB patients and family members, and manage TB patients through routine household visits [9].

To explore the feasibility, yield, and cost of active case-finding (ACF) among seniors in rural China, we conducted a pilot project in a small rural community in Sichuan Province from March to June 2017. We added TB screening to annual clinic-based check-ups offered through the national public health service program for seniors. Findings from this project can be used to strengthen the TB program in China as well as programs in other countries with aging populations and a high burden of TB disease.

## Methods

### Project location and population characteristics

We piloted ACF among seniors in Bayi, a township in Jiangyou County, Sichuan Province. In 2016, Bayi had a population of 17,323. Approximately 14% of the population (2,400 persons) were seniors over 65 years of age [10]. Bayi residents were primarily employed as migrant workers or farmers and had a mean annual income of $2,330. At the time of the project, the registration rate for TB in Bayi was 45.8 cases per 100,000 people, including four TB cases among seniors. This was lower than the national registration rate of 60.5 cases per 100,000 people for the same period. We selected Bayi as a pilot site based on qualitative factors primarily related to support from the township and Jiangyou County officials. Other factors included the availability of TB diagnostic testing at the Jiangyou County Center for Disease Control and Prevention (CDC), and the capacity to identify and enroll seniors to participate in the project.

### TB case definitions

We defined suspected TB as a patient presenting with chronic cough lasting ≥ 2 weeks or hemoptysis [11]. Laboratory-confirmed TB was defined as a patient with:

1) Smear-positive pulmonary TB (PTB) (presence of two or more positive smears, one positive smear plus an abnormal chest X-ray (CXR) result consistent with TB, or one positive smear plus a positive culture; or
2) Negative smear, but culture-positive for TB.

A clinically diagnosed PTB case was defined by the presence of three negative smears and CXR abnormalities consistent with active PTB in a patient meeting at least one of the following criteria:

1) Clinical TB symptoms (chronic cough or hemoptysis); or
2) Strong positive purified protein derivative (PPD) reaction (average diameter of induration ≥15 mm, or <15 mm but with any of the following locally: double-circle, blister, necrosis, lymphangitis); or
3) TB lesions confirmed by histopathological examination of extrapulmonary tissues.

Patients with three negative smears and other pulmonary diseases excluded by diagnostic treatment or follow-up observations were also defined as clinically diagnosed PTB. All diagnoses were made in accordance with National TB Guidelines based on clinical, microbiological, and radiological evidence [11].

### Participant recruitment

All seniors ≥65 years of age residing in Bayi Township were eligible to participate in the active TB case-finding pilot project. A list of all eligible senior residents was obtained from the local public security station per the approved project protocol. The list included the name, age, and place of residence of each senior in Bayi.

We defined the following as risk factors for TB: previous TB diagnosis, HIV infection, close contact with smear-positive TB patients, diabetes mellitus, work history as a miner, body mass index (BMI) <18.5, chronic obstructive pulmonary disease, use of immunosuppressant drugs, tobacco use, or heavy drinking (definitions are included in **Table 1**). We developed our initial list of risk factors based on findings from systematic literature reviews of TB risk-factors conducted by WHO and the Chinese Center for Disease Control and Prevention [12,13]. We then supplemented this list using findings from a large population based study implemented in China in 2013, in which individual risk factors and TB disease status were collected and analyzed from a population of seniors [14]. We included chronic obstructive pulmonary disease and the use of immunosuppressant drugs to examine their effectiveness as additional TB screening factors in this pilot project.

**Table 1:** Definition of TB symptoms and risk factors used for active TB case finding in Bayi Township, China from March–June 2017.

Factors	Definition
TB symptoms	Chronic cough ≥2 weeks or hemoptysis
Previous TB cases^*^^§^	PTB patients who finished TB treatment or cured
HIV/AIDS^*^^¶^	Registered in local CDC database
Close contacts^*^^¶^^§^	People in direct contact with newly registered sputum smear positive PTB patients (on initial treatment and retreatment), including family members, colleagues and schoolmates of PTB patients
Diabetes Mellitus^*^^¶^^§^	In the *Residents Health Records*, the disease history showed "Diabetes Mellitus", or Fasting blood-glucose records showed ≥7.0mmol/L
Miners^*^^¶^	In the *Residents Health Records*, the record of occupational exposure showed "exposed to dust"
BMI<18.5^*^^¶^^§^	Weight (kg)/Height^2^ (m^2^) <18.5
Chronic obstructive pulmonary disease^ŧ^	In the *Residents Health Records*, the disease history showed "Chronic obstructive pulmonary disease"
Immunosuppressant use^ŧ^	In the *Residents Health Records*, the current medications showed "using immunosuppressant"
Tobacco use^*^^¶^^§^	In the *Residents Health Records*, the lifestyle part showed "previously smoking" or "currently smoking"
Heavy drinker^*^^¶^^§^	Drinking more than one unit (41 grams pure alcohol for male, 21 grams pure alcohol for female) per day

* Risk factors used for active TB case finding based on reference 13.

¶ Risk factors used for active TB case finding based on reference 12.

§Risk factors used for active TB case finding based on reference 14.

ŧ Included as additional TB screening factors in this pilot project.

To identify seniors with previous TB disease, we cross-referenced the list of eligible senior residents with the National TB Information Management System (TBIMS) for patients diagnosed with TB since 2005. We reviewed the health records of all seniors to identify TB cases diagnosed before deployment of TBIMS in 2005. Jiangyou CDC staff provided information regarding seniors in Bayi living with HIV/AIDS. A list of elderly close contacts of smear-positive pulmonary TB patients diagnosed from September 2016 to March 2017 was obtained from TBIMS. We captured information on the other TB risk factors during the health record reviews.

### Screening procedures

Annual check-ups for seniors were held at village clinics and the Bayi township hospital from March to June 2017. Seniors were informed of the dates and times of the check-ups from village doctors. During the annual physical check-ups, project staff administered a written questionnaire to obtain information on TB symptoms. Seniors displaying clinical symptoms or having at least one of the ten risk factors were offered a follow-up CXR. Seniors with an abnormal CXR or clinical symptoms (if CXR was normal) were asked to provide sputum samples for laboratory diagnosis using smear and solid culture. We collected one sputum sample onsite and provided participants with collection containers for two additional sputum samples (one for the night and one for the following the morning). Primary HCWs from the village clinic collected sputum samples from the participants’ homes and delivered these to Bayi township hospital. Jiangyou CDC staff transported available samples from the hospital to the CDC laboratory for testing once a day. Laboratory staff documented CXR and sputum test results on a paper form that included the patient’s name and project ID.

We conducted home visits to administer the project questionnaire about TB symptoms to seniors who missed the scheduled follow-up appointment. Seniors with at least one of the ten risk factors were referred to the local township hospital for a CXR. Seniors who were referred but missed the scheduled CXR were contacted by telephone and encouraged to complete the examination. If a senior was still unable to complete the CXR appointment, the local township hospital arranged transportation (via ambulance) to help the senior receive the recommended CXR. We documented reasons for non-participation and loss to follow up.

### Costs associated with ACF

We recorded all costs associated with the pilot ACF project not covered by the Basic Public Health Service Program. This included labor and material costs for health record reviews, household visits, phone call surveys, transportation of participants and samples, CXR diagnostic tests, and sputum acid-fast bacilli microscopy and culture. Time spent by project staff on conducting household visits and participant follow-up was recorded using a standard form. The average daily salary (8 hours per day) was used to calculate human costs. Although we did not collect project costs from seniors, we anticipate that these were primarily linked to opportunity costs (i.e., time away from other activities) and were likely to be minimal.

### Qualitative evaluation

We conducted in-depth individual interviews with seven local project health staff from Jiangyou CDC (n = 3), the TB-designated hospital in Jiangyou (n = 2), and the Bayi township health center (n = 2). We also organized four focus group sessions for health workers from the Bayi township health center and 10 village clinics. Each focus group included four health workers. The qualitative questionnaire for health staff interviews and focus group sessions centered on four areas: 1) the importance of TB in the community, 2) the convenience of TB screening among seniors from a HCW perspective, 3) the feasibility of including TB screening in the Basic Public Health Service Program, and 4) recommendations for TB screening in the future. Two national level project staff (Zhang and Cheng) conducted the in-depth interviews and facilitated the focus group sessions. We held the interviews and focus group sessions at the Bayi township hospital. Interviews and focus group sessions were completed in 15 minutes and 45 minutes, respectively.

The same two national level project staff also conducted in-depth individual interviews with 20 randomly selected seniors, including 10 with suspected TB (i.e., those who presented with CXR abnormalities consistent with TB during screening). The questionnaire for seniors focused on three areas: 1) convenience of the TB screening activities, 2) reasons for wanting to participate in TB screening activities the following year, and 3) recommendations for TB screening in the future. We also held the senior interviews at Bayi township hospital. Responses from interview and focus group discussions were documented paper survey forms and entered into an electronic word document for review and analysis.

### Statistical analysis

We entered previous diagnoses, screening questionnaire data, and laboratory results collected from each senior in Bayi into an EpiData database (EpiData Association, Odense, Denmark). Reasons for non-participation were also linked to the questionnaire data when possible. Data were imported into SAS 9.3 (SAS Institute Inc., Cary, NC, USA) for statistical analysis. We calculated the participation rate as the number of seniors participating in the TB screening project divided by the total number of eligible seniors residing in Bayi at the time of the project. We calculated the TB detection rate as the number of seniors diagnosed with active TB per 100,000 seniors screened. Descriptive statistics were generated for patient characteristics, prevalence of each of the ten risk factors, and reasons for non-participation.

Total project costs and cost per TB case diagnosed were calculated using Microsoft Excel (Microsoft Corporation, Redmond, WA, USA) in United States dollars. A thematic framework analysis was used to summarize the qualitative interviews. Two project staff (Zhang and Xia) independently reviewed and assigned qualitative responses for each main topic area to themes using the constant comparative method. This method identified major themes by comparing one segment of responses with another to identify similarities and differences [15]. Consensus about emerging themes was reached among the authors following discussion. Individual responses were subsequently classified into one of these major themes.

### Ethical considerations

The pilot project protocol was reviewed and approved by the Institutional Review Board of the Chinese Center for Disease Control and Prevention (Serial No: 201612). The United States Centers for Disease Control and Prevention approved the project through a routine program evaluation. Written informed consent was obtained from seniors before screening and post-screening interviews, and verbal informed consent was obtained from project staff before the in-depth interviews and focus group discussions. China’s national diagnostic criteria (Ministry of Health, 2008) were applied to identify TB patients during this project. All diagnosed patients were registered, treated, and managed according to national guidelines. Personal identifying information (PII) collected during the project was maintained in a secure and/or password-protected location and was removed from the project database prior to analysis.

## Results

### Participant characteristics and TB case-finding

**Fig 1:**
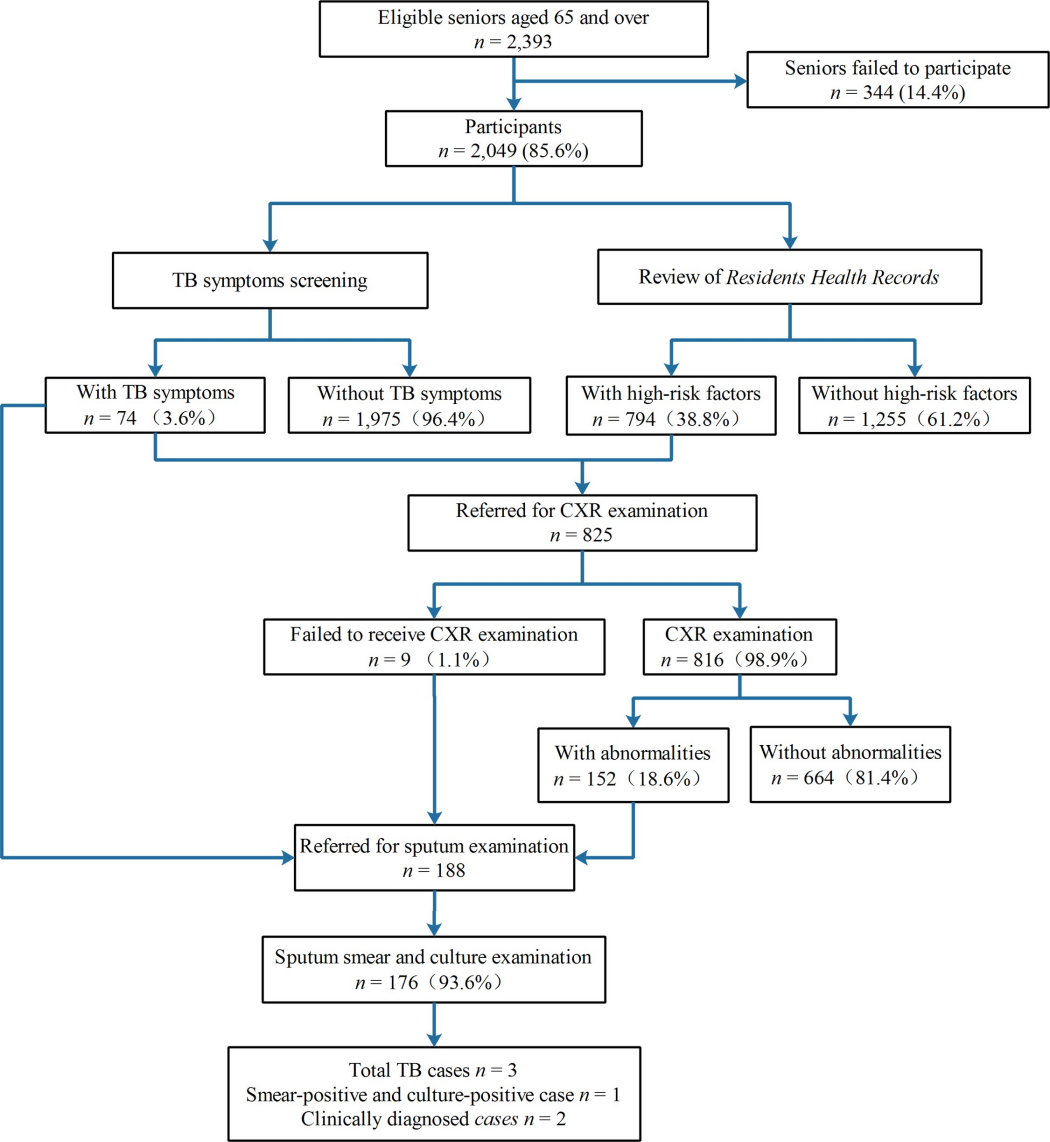
Schematic diagram of the study design and data collection in a pilot active case finding project among seniors aged ≥65 years in Bayi township, Sichuan Province, China, March–June 2017. TB: tuberculosis; CXR: chest x-ray.

Between March and June 2017, 2,049 (85.6%) of the 2,393 seniors residing in Bayi participated in the TB pilot project. Of these, 1,315 (64.2%) and 734 (35.8%) were screened at a local health facility or during home visits, respectively. The median age was 72 years (interquartile range: 68–77), and 48.4% were male. Of the seniors screened, 794 (38.7%) presented with at least one of the 10 TB risk factors, and 74 (3.6%) had symptoms consistent with active TB (**Fig 1 and Table 2**). Forty-three seniors (2.1%) presented with at least one risk factor and symptoms consistent with active TB.

**Table 2:** Demographic characteristics and risk factors of seniors aged ≥ 65 years participating in the active case finding project by TB symptoms and diagnosis, Bayi township, Sichuan Province, China, March–June 2017.

Characteristics	N (%)	Presenting with TB Symptoms (%)	Diagnosed with TB¶ (%)
**All**	2,049 (100.0)	74 (3.6)	3 (0.2)
**Sex**			
Male	992 (48.4)	38 (3.8)	1 (0.1)
Female	1,057 (51.6)	36 (3.4)	2 (0.2)
**Age group (years)**			
65–74	1,311 (64.0)	46 (3.5)	2 (0.2)
75–84	614 (30.0)	27 (4.4)	1 (0.2)
≥ 85	124 (6.0)	1 (0.8)	0 (0.0)
High-risk factors*			
None	1,255 (61.3)	31 (2.5)	0 (0.0)
Tobacco use	442 (21.6)	23 (5.2)	1 (0.2)
Alcohol use	332 (16.2)	11 (3.3)	1 (0.3)
Chronic obstructive disease	200 (9.8)	13 (6.5)	2 (1.0)
Diabetes mellitus	186 (9.1)	11 (5.9)	0 (0.0)
BMI < 18.5	22 (1.1)	1 (4.6)	0 (0.0)
HIV/AIDS	2 (0.1)	1 (50.0)	0 (0.0)
Occupation as miner	1 (< 0.1)	0 (0.0)	0 (0.0)
Close contact with TB	0 (0)	0 (0.0)	0 (0.0)
Immunosuppressant treatment	0 (0)	0 (0.0)	0 (0.0)

*Participants can have more than one high-risk factor

¶Percent calculated from total participating in screening and receiving recommended examinations (i.e., excludes 7 seniors not receiving chest x-ray or providing sputum samples)

Of the 825 seniors referred for CXRs (i.e., those presenting at least one risk factor and/or TB symptoms), 816 (98.9%) completed the additional examination. Of these, 188 (23%) were referred for diagnostic testing, and 176 (93.6%) completed the sputum examination. Three were diagnosed with active TB, including one smear-positive and culture-positive confirmed case and two clinically diagnosed cases. Of the three seniors with active TB, two were female and one was male (aged 65, 73, and 81, respectively). None reported clinical TB symptoms. The two clinically diagnosed cases had CXR abnormalities consistent with TB and positive reactions to the PPD skin test. According to the health record review, the 81-year-old male had a history of alcohol consumption and smoking, and the two female cases (aged 65 and 73 years) had chronic obstructive pulmonary disease. The overall detection rate or yield was 146 TB cases per 100,000 seniors screened. The detection rate in seniors with at least one of the 10 TB risk factors was 378 TB cases per 100,000 seniors screened.

During the 4-month project period, 344 (14.4%, 344/2393) seniors did not participate in the initial check-up. Of the seniors providing a reason for not participating, 245 (71.2%) no longer lived at the address on file, 69 (20.1%) had limited confidence in the examinations, 16 (4.6%) reported mobility problems, and 10 (2.9%) reported not having enough time. Four seniors did not provide a reason.

### Project costs

The total cost of the project was $14,692, and the cost for each newly detected TB case was $4,897 (**Table 3**). Total project costs included $390 for health records review, $360 for household visits, and $23 for follow-up telephone calls. CXRs; transportation of sputum specimens, sputum smears, and cultures; and transportation of seniors also contributed to the project’s costs.

**Table 3:** Costs of active case finding among seniors aged ≥ 65 years not covered by the *Basic Public Health Service Program* in Bayi township, Sichuan Province, China, March–June 2017.

Activities	Calculation	Cost (US$)	% of Total Cost
Review of Senior Residents Health Records	12 staff × 13 hours × $2.5 /hour	390	2.6
Household investigation of seniors unable to attend scheduled health examination	144 hours × $2.5/hour	360	2.4
Phone call survey	6 hours × $2.5/hour + 6 hours * $0.02/minute (telephone charge) × 60 minutes/hour	22	0.2
Chest x-ray examination	2 staff × 32 days × $20/day + 816 participants × 2 films × $4.853/film	9,200	62.6
Transportation of sputum specimens	15 times × $13.24/time	199	1.4
Sputum smear test	176 participants × $3.1/person (smear charge) + 176 participants × $2.2/person (subsidy for staff)	932	6.3
Sputum culture test	176 participants × $4.71/person (culture charge) + 176 participants × $2.94/person (subsidy for staff)	1,346	9.2
Arranging ambulance transportation for seniors to receive the chest x-ray examination	1023 km (ambulance mileage) × 33 L/100km (Fuel consumption) × $0.956 /L + 3 staff × 32d days × $20/day	2,243	15.3
**Total**	**$14,692**		**100**

### Qualitative evaluation

During our in-depth interviews and focus group discussions, most project staff and health care workers provided positive feedback regarding the aims of the project. Major themes identified and examples from interviews and discussions for each topic area are presented in **Table 4**. For example, in response to feedback on the TB ACF project, one staff member stated that ACF “…is convenient and feasible for residents…” and it “…arranges x-ray and sputum examinations for high-risk populations.” At the same time, workers expressed concerns about whether TB screening should be permanently included as part of annual check-ups due to the time required for home visits, CXRs, and sputum tests. One worker indicated that there is a “deficiency [in] human resources…leading to excess burden and time conflicts…with no additional reward…” However, most workers supported the implementation of the project if more resources and staff could be provided.

**Table 4:** Feedback from public health staff and health care workers on active case finding among seniors aged ≥ 65 years captured through in-depth interviews and focus groups, Bayi township, Sichuan Province, China, March–June 2017*.

Topic area	Theme	Example response
Importance of TB	Prioritization of infectious disease	*It is an important public health issue*, *as an airborne infectious disease*, *the control of which is required and assigned by the state*, *in consideration of its high drug resistance*, *occurrence in low-income population*, *potential to cause discrimination against patients*, *great subsequent damage to health of patients*, *lack of prevention and control measures*, *and lack of knowledge in the public*
Active case finding for seniors	Convenient	*It is convenient and feasible for the residents; it increases the number of inspection items*, *with high acceptance; and it arranges X-ray examination and sputum examination for high-risk population*.
	Not convenient	*It is difficult by taking into account that (1) the elderly have to complete a lot of physical examinations*, *and the compliance with the requirements cannot be guaranteed; (2) for suspicious symptoms*, *the consultation process is too complicated*, *in addition to the Basic Public Health Service Program*, *which already includes a number of questions; and (3) it is difficult to complete the X-ray examination*, *because subjects usually need to wait for a long time*, *until a specified number of subjects have been assembled*, *then they will be transported by the ambulance for the examination*.
Include TB screening in Basic Public Health Service Program	Feasible	*It is feasible*, *but the chest X-ray examination should be replaced by digital radiography; it should not be limited to the elderly*, *but open to all individuals with high risk factors*, *since there are many young patients with tuberculosis; and most people pay attention to health problems*, *and chest X-ray examination can also find other diseases*.
	Not feasible	*Infeasible because of deficiency in human resources*, *with only 2 full-time workers; and the staffing for X-ray examination is inadequate*, *leading to excessive burden and time conflicts; the workload has been increased significantly in a short time*, *with no additional reward provided; The quality and ability of X-ray examination and diagnosis needs to be improved at the township level*.
Recommendations	Increase case finding	*Elderly people with high risk factors should get the chest X-ray examination*, *such as smoker*, *drinker and one with weight loss*, *once in each year*, *combined with the basic public health program*. *For others*, *the program should only focus on increasing health awareness*. *Patients with TB disease will be included into the treatment procedure*.
	Required resources	*Increase the X-ray examination equipment (computed radiography*, *digital radiography)*, *enhance the personnel input*, *improve the radiograph review ability or organize experts to review radiographs; arrange vehicles specifically for picking up seniors to take them to the X-ray examination; increase funds*, *including physical examination funds and staff allowances*, *mobilize the enthusiasm; get support and approval from the township government*, *as well as the support from the village head*.

*Includes feedback from seven in-depth interviews and four focus group discussions with 16 health workers.

Almost all 20 interviewed seniors supported the project. Major themes and examples for each topic area are presented in **Table 5**. One senior stated, “the [TB] disease could be diagnosed earlier, thus could be treated earlier; there is no such opportunity in normal times, really a rare chance.” Most seniors indicated that having CXRs and testing performed at the same time and in the same location avoided additional clinic visits and was very beneficial. For example, according to a few seniors, the “process is convenient, and not far away from home. [It] feels convenient whether or not there is a pick-up vehicle.”

**Table 5:** Feedback from seniors on perceptions of active case finding project captured through in-depth interviews, Bayi township, Sichuan Province, China, March–June 2017*.

Topic area	Theme	Example response
Active case finding for seniors	Convenient	*More examinations were available this year*, *and the pick-up vehicle makes it easy; process is convenient*, *not far away from home; it feels convenient whether or not there is a pick-up vehicle*.
	Perceived benefit	*The disease can be diagnosed earlier*, *thus can be treated earlier; there is no such opportunity at other times*, *really a rare chance; X-ray examination has been added to make the examination program more comprehensive*, *which can be used to check whether the patient has got any type of lung problem [in addition to TB]*.
Include TB screening in Annual Basic Public Health Service Program Check-ups	Will participate	*I will participate in the activities*, *because they are good for the health*, *and some people did not participate just because they had farm work to do*, *or were not suitable to do specific test because of having meal; I will accept it as the support from the state*, *the program can diagnose and treat the disease earlier; my family supports this*.
	Will seek treatment	*I may choose to get it treated*, *depending on the economic situation; I will get it treated definitely*, *because any disease should be treated if found; I will take further examinations to confirm the diagnosis*, *and accept the medication treatment earlier to make it controlled at the early stage; if the diagnosis of TB has been confirmed*, *I will accept the treatment at my cost; and if the diagnosis of TB is not confirmed*, *I will not accept the treatment*.
Recommendations	Increase resources to Township	*The equipment in the township health center is inadequate*, *and it needs to add CT equipment; the construction of township health center is needed*, *saving the need of patients to go to the county hospital; the pick-up vehicles could not be driven into the village due to narrow roads*, *so it would be better if there was somebody to help the elderly get on the vehicles; patients identified with TB disease after the examination should receive treatment free of charge*.

*Includes feedback from 20 seniors participating in the active case-finding project

## Discussion

In this pilot project, we incorporated active TB case-finding with annual senior medical check-ups supported through the Basic Public Health Service Program. By screening for TB symptoms and high-risk factors, we identified three active TB cases. All three were successfully referred for treatment. The estimated detection rate was 146 TB cases per 100,000 seniors screened. Although lower than expected (based on a prevalence of 502 TB cases per 100,000 seniors in China) [16], ACF aims to identify and treat persons with TB who would not otherwise have sought treatment for their symptoms [17,18]. This pilot project demonstrated feasibility based on the capacity of local health care staff to implement the project and a participation rate of 85.6% among the senior population. However, additional assessments are needed elsewhere in China to evaluate strategies to reduce project costs and further refine our TB screening criteria.

For this pilot ACF project, we incorporated TB symptom and risk factor screening into the existing annual check-up program for seniors [9]. This approach benefited from using an established health check-up platform while minimizing additional burden on local health care staff. During follow-up interviews, seniors indicated that access to free transportation to the clinic for check-ups and screening were the most positive aspects of the project. This is supported by the large percent of seniors (64.2%) in Bayi who participated in the clinic-based TB screening pilot project. The clinic-based approach can also strengthen the integration of health care service for seniors through the Basic Public Health Service Program and help detect other non-TB related conditions. TB screening during annual check-ups differs from door-to-door [19] or population-based [16] approaches which rely on substantial logistic and human resources for implementation. Conducting ACF using door-to-door approaches requires project staff to visit all individual households, often through numerous repeat visits, to identify and enroll eligible members of the target population [17]. The validity of population-based approaches typically depends on the accurate use of multi-stage cluster sampling designs, resulting in longer travel distances and more complex statistical analyses [16]. Both approaches would have likely required more time and resources if used in Bayi, possibly resulting in the same yield.

Other active TB case-finding projects have focused ACF among close contacts of smear-positive TB cases, prisoners, and HIV/AIDS patients [20–22]. The effectiveness of these projects commonly relied on a high TB disease burden in a well-defined target population, either in a specific setting (prisons [21]) or in a known risk group (close contracts [20] or persons with a HIV/AIDS [22]). Within each setting or risk group, project staff most often screened for TB symptoms, such as chronic cough or hemoptysis. For example, an ACF project in Uganda identified 39 active TB cases among 199 persons reporting a chronic cough [23]. In this setting–also a high HIV burden area—symptom screening alone could be used efficiently in ACF projects to detect persons with TB disease. In Bayi, however, none of the three detected TB cases presented with a chronic cough.

Screening for risk factors is likely more useful than symptom screening for identifying active TB cases among the senior population. Although approximately 54.9% of seniors with active TB present with chronic cough [24], most seniors, particularly elderly females [23], do not seek health care due to their symptoms, resulting in undetected cases and transmission. Ideally, ACF could incorporate both symptom and risk factor screening along with molecular testing. This comprehensive approach is resource intensive and would be impractical in many locations in high TB endemic communities. To reduce the resource burden on health care services, we relied on a list of 10 risk factors for active TB case screening. This list was developed using previously published literature reviews [12, 13] well as results from a large cohort study on TB in China [14]. We included chronic obstructive pulmonary disease and use of immunosuppressant drugs as supplemental risk factors. A total of 825 seniors were referred for CXRs; most seniors reported individual or combined use of tobacco, alcohol, and chronic obstructive disease. Two of the three detected TB cases had chronic obstructive pulmonary disease, supporting the potential use of this condition as a risk factor for TB screening. A smaller percentage of all seniors had a previous diagnosis of diabetes mellitus. Close contact, malnutrition, and diabetes are known risk factors for active TB disease [12,13,20], but were rare in our pilot project population. None of the seniors reported use of immunosuppressant drugs. These findings may reflect some geographic variability in the optimal screening criteria. In resource limited communities, TB program staff may need to adapt the ACF screening criteria to better reflect the local TB epidemiology. Additional ACF projects should be implemented elsewhere in China, including in high TB prevalence areas, to further evaluate the findings from this pilot project. In all locations, risk factor screening will probably be more effective than use of symptoms alone for ACF among seniors.

At the same time, additional training for health care workers on identifying and diagnosing active TB could also be useful since only 188 of the 825 screened seniors were referred for CXRs. (i.e., we were unable to verify the application of the screening and testing procedures and interpretation). Clinical training is particularly important for detecting active TB among seniors with negative sputum results. Health care providers in Bayi township may be less familiar with the use of TB clinical diagnosis criteria than those in areas with a higher prevalence of TB. Similar trainings could also be useful for the routine detection of TB disease among seniors in other low TB prevalence communities.

Our analysis suggests that the cost of detecting a single TB case through ACF among seniors was $4,897. We estimate that more than 62% ($9,200/$14,692) of the pilot project costs were directly attributable to CXRs. If this examination could be included as part of the Basic Public Health Service Program, then the cost of ACF could be reduced to $1,831 or less, for each TB case detected in Bayi township. ACF project costs could also be off-set with expenses saved for each detected TB case in terms of morbidity and mortality, potential transmission, and general social and economic well-being of the senior population [25]. Each untreated active TB case in the senior population, for example, could lead to increased costs for care and hospitalization, or TB transmission to other household and community members, potentially leading to new active TB cases. Adjusting overall ACF project costs to reflect ‘predicted savings’ would likely substantially lower the cost per TB case detected [26,27]. ACF can also help seniors identify non-TB related lung conditions, also a potential cost-savings benefit.

Implementation of this pilot project involved several limitations. First, we relied on information provided by the local security office to identify eligible seniors, which was likely not up-to-date. We were unable to determine the number of seniors new to the area who could have been missed. A simple census along with community outreach on TB health education and the benefits of TB screening could be conducted prior to implementing ACF activities. Second, we used “work history as a miner” as a risk factor based on information from personal health records of the Basic Public Health Service Program. Only one senior (of the 2,049 screened) reported previous work as a miner; however, information on duration of work exposure was not recorded, limiting the usefulness of this risk factor for TB screening. Third, we relied on tests having either limited sensitivity (sputum smear microscopy) or specificity (CXR), resulting in possible diagnostic errors [28,29]. This could have potentially resulted in missed TB cases despite TB screening activities. The use of new diagnostic tools, such as GeneXpert, could help mitigate these diagnostic challenges. Finally, this was a small pilot project, and our findings are unlikely to be representative of other locations in China. Findings from this pilot project should be combined and interpreted collectively with other ACF projects before implementing TB ACF among seniors on a larger scale.

## Conclusions

Our pilot project on active TB case finding among seniors detected three previously unidentified TB cases. Although the case-finding yield was low, we demonstrated feasibility of integrating ACF with annual check-ups supported through the Basic Public Health Service Program. Targeting seniors with at least one known TB risk factor could help detect previously unidentified TB cases. However, similar projects in communities with a higher TB prevalence are needed to further evaluate the yield and required resources prior to implementation on a larger scale. Findings from our pilot project should be combined with data from future ACF projects to improve TB screening criteria. An adaptable and flexible ACF approach may be needed to address geographic differences in TB prevalence, population demographics, and available human resources.

### Disclaimer

The authors alone are responsible for the views expressed in this paper. These views do not necessarily represent the decisions, policies, or views of the United States Centers for Disease Control and Prevention.
